# Canonical Poly(A) Polymerase Activity Promotes the Decay of a Wide Variety of Mammalian Nuclear RNAs

**DOI:** 10.1371/journal.pgen.1005610

**Published:** 2015-10-20

**Authors:** Stefan M. Bresson, Olga V. Hunter, Allyson C. Hunter, Nicholas K. Conrad

**Affiliations:** Department of Microbiology, University of Texas Southwestern Medical Center, Dallas, Texas, United States of America; Aarhus University, DENMARK

## Abstract

The human nuclear poly(A)-binding protein PABPN1 has been implicated in the decay of nuclear noncoding RNAs (ncRNAs). In addition, PABPN1 promotes hyperadenylation by stimulating poly(A)-polymerases (PAPα/γ), but this activity has not previously been linked to the decay of endogenous transcripts. Moreover, the mechanisms underlying target specificity have remained elusive. Here, we inactivated PAP-dependent hyperadenylation in cells by two independent mechanisms and used an RNA-seq approach to identify endogenous targets. We observed the upregulation of various ncRNAs, including snoRNA host genes, primary miRNA transcripts, and promoter upstream antisense RNAs, confirming that hyperadenylation is broadly required for the degradation of PABPN1-targets. In addition, we found that mRNAs with retained introns are susceptible to PABPN1 and PAPα/γ-mediated decay (PPD). Transcripts are targeted for degradation due to inefficient export, which is a consequence of reduced intron number or incomplete splicing. Additional investigation showed that a genetically-encoded poly(A) tail is sufficient to drive decay, suggesting that degradation occurs independently of the canonical cleavage and polyadenylation reaction. Surprisingly, treatment with transcription inhibitors uncouples polyadenylation from decay, leading to runaway hyperadenylation of nuclear decay targets. We conclude that PPD is an important mammalian nuclear RNA decay pathway for the removal of poorly spliced and nuclear-retained transcripts.

## Author Summary

Cells control gene expression by balancing the rates of RNA synthesis and decay. While the mechanisms of transcription regulation are extensively studied, the parameters that control nuclear RNA stability remain largely unknown. Previously, we and others reported that poly(A) tails may stimulate RNA decay in mammalian nuclei. This function is mediated by the concerted actions of the nuclear poly(A) binding protein PABPN1, poly(A) polymerase (PAP), and the nuclear exosome complex, a pathway we have named PABPN1 and PAP-mediated RNA decay (PPD). Because nearly all mRNAs possess a poly(A) tail, it remains unclear how PPD targets specific transcripts. Here, we inactivated PPD by two distinct mechanisms and examined global gene expression. We identified a number of potential target genes, including snoRNA host genes, promoter antisense RNAs, and mRNAs. Interestingly, target transcripts tend to be incompletely spliced or possess fewer introns than non-target transcripts, suggesting that efficient splicing allows normal mRNAs to escape decay. We suggest that PPD plays an important role in gene expression by limiting the accumulation of inefficiently processed RNAs. In addition, our results highlight the complex relationship between (pre-)mRNA splicing and nuclear RNA decay.

## Introduction

Eukaryotic messenger RNAs (mRNAs) undergo a series of maturation events before they are exported to the cytoplasm and translated. The complexity of alternative processing increases the likelihood of mistakes that produce aberrant mRNAs encoding defective proteins. In addition, pervasive transcription occurs across nearly the entire mammalian genome resulting in the generation of nonfunctional RNAs. Consequently, cells have evolved RNA quality control (QC) pathways to eliminate these RNAs [1,2].

The best-characterized RNA QC pathway is nonsense-mediated mRNA decay (NMD)[3]. NMD targets cytoplasmic mRNAs with premature termination codons (PTCs), a potentially dangerous class of RNAs that produce truncated and possibly dominant-negative proteins. NMD is limited in at least three important ways. First, NMD recognizes PTC-containing transcripts upon translation, so each defective transcript still produces one polypeptide. This could be harmful to cells for highly transcribed NMD targets or particularly toxic polypeptides. Second, NMD is stimulated by the presence of a splice junction to identify PTCs, so transcripts from intronless genes will generally not be recognized. Third, pervasive transcription produces nuclear transcripts that would not be targeted by the cytoplasmic NMD machinery.

Cells have additional nuclear RNA QC pathways to degrade RNAs not targeted by NMD, but the mechanisms involved remain unclear. Recently, functions for the nuclear poly(A) binding protein PABPN1 in RNA decay has been reported [4–6]. An RNA-seq study showed that knockdown of PABPN1 increases the accumulation of endogenous long noncoding RNAs (lncRNAs), several noncoding snoRNA host genes (ncSNHGs) and transcripts upstream of mRNA gene promoters [4]. In addition, the Kaposi’s sarcoma-associated herpesvirus (KSHV) produces an abundant polyadenylated nuclear (PAN) RNA during the lytic phase of viral infection. A cis-acting element, called the ENE, protects PAN RNA from PABPN1-mediated decay by forming a triple helix with the poly(A) tail [5,7,8]. PABPN1 additionally promotes the degradation of a poorly exported intronless β-globin mRNA, but not its spliced and efficiently exported counterpart, suggesting it serves a QC function for non-exportable polyadenylated RNAs. PABPN1-mediated decay has been observed in *S*. *pombe* and humans suggesting an important conserved function [9–12].

The canonical mammalian poly(A) polymerases PAPα and PAPγ (PAP), and the nuclear exosome are involved in PABPN1-mediated decay of intronless β-globin and PANΔENE reporters [5]. Several observations demonstrate that hyperadenylation by PAP promotes decay. First, knockdown of either PABPN1 or PAP stabilizes RNAs with shorter poly(A) tails. Second, knockdown of the exosome leads to the accumulation of hyperadenylated products. Third, inhibition of polyadenylation by cordycepin inhibits RNA decay. Fourth, expression of a dominant-negative PABPN1 double point mutant (L119A/L136A or LALA) that binds RNA but cannot stimulate PAP [13] stabilizes target RNAs. A global decay function for PAP is validated by the analyses reported here, so we now refer to this pathway as PABPN1 and PAPα/γ**-**mediated RNA decay (PPD).

PABPN1 and PAP have been extensively characterized for their roles in mRNA 3´-end formation [14]. Polyadenylation is initiated by co-transcriptional recruitment of the cleavage and polyadenylation specificity factor (CPSF) to the AAUAAA polyadenylation signal (PAS) through the CPSF30 and WDR33 subunits [15,16]. Extensive in vitro studies defined the roles of PAP, PABPN1, and CPSF in the normal polyadenylation of mRNA 3´-ends [13,17]. Without CPSF, PAP has low binding affinity for RNA, but the CPSF-PAP interaction drives binding and generation of an oligo(A) tail. PABPN1 binds the oligo(A) tail and forms a complex with PAP-CPSF-oligo(A). PAP becomes tightly tethered to the RNA, and polyadenylation is highly processive to ~200–300 nt poly(A) length. At this point, the interaction between PAP and CPSF is lost and polyadenylation becomes distributive, but this distributive polyadenylation continues to be stimulated by PABPN1.

We proposed that PABPN1-dependent and CPSF-independent stimulation of distributive PAP activity provides the polyadenylation associated with PPD [5]. Here, we refer to this as “hyperadenylation” as it occurs after the initial 3´-end formation step. To explore this globally, we performed RNA-seq following inactivation of hyperadenylation by two distinct methods. Consistent with the PABPN1 knockdown studies, we found that several classes of lncRNAs, including ncSNHGs, primary microRNA transcripts, and upstream antisense RNAs, are susceptible to PPD. In addition, we identified mRNAs and (pre-)mRNAs with retained introns that are PPD targets. Surprisingly, transcription inhibition led to a robust PABPN1-dependent hyperadenylation of a large pool of nuclear RNAs apparently due to the uncoupling of hyperadenylation from decay. Finally, we observed that a CPSF-independent poly(A) tail initiates PPD, but hyperadenylation was not sufficient for PPD in the absence of PABPN1. From these studies, we conclude that PPD is a major human nuclear RNA decay pathway.

## Results

### Identification of PPD targets

We aimed to generate a high-confidence list of PPD targets by performing RNA-seq on polyadenylated RNA from cells in which PPD-associated hyperadenylation had been inactivated by two independent methods. For one treatment, we prepared RNA from cells after a three-day co-depletion of PAPα and PAPγ by siRNAs (siPAP)(S1A Fig). For the second treatment, we created a stable cell line expressing myc-tagged LALA under control of a tetracycline-responsive promoter (TetRP). Following a three-day induction of LALA, we collected RNA in preparation for high-throughput sequencing. Under these conditions, LALA was expressed at levels only slightly greater than endogenous wild-type PABPN1 (S1A Fig). We examined polyadenylated RNAs from detergent-insoluble nuclear fractions of control, LALA, and siPAP-treated cells and on total polyadenylated RNA from control and siPAP-treated cells. Our fractionation procedure enriches for chromatin and nuclear speckle-associated RNAs [18–20]. Admittedly, the protocol results in the loss of some detergent-soluble nuclear material, but the fractions have little cytoplasmic contamination.

**Fig 1:**
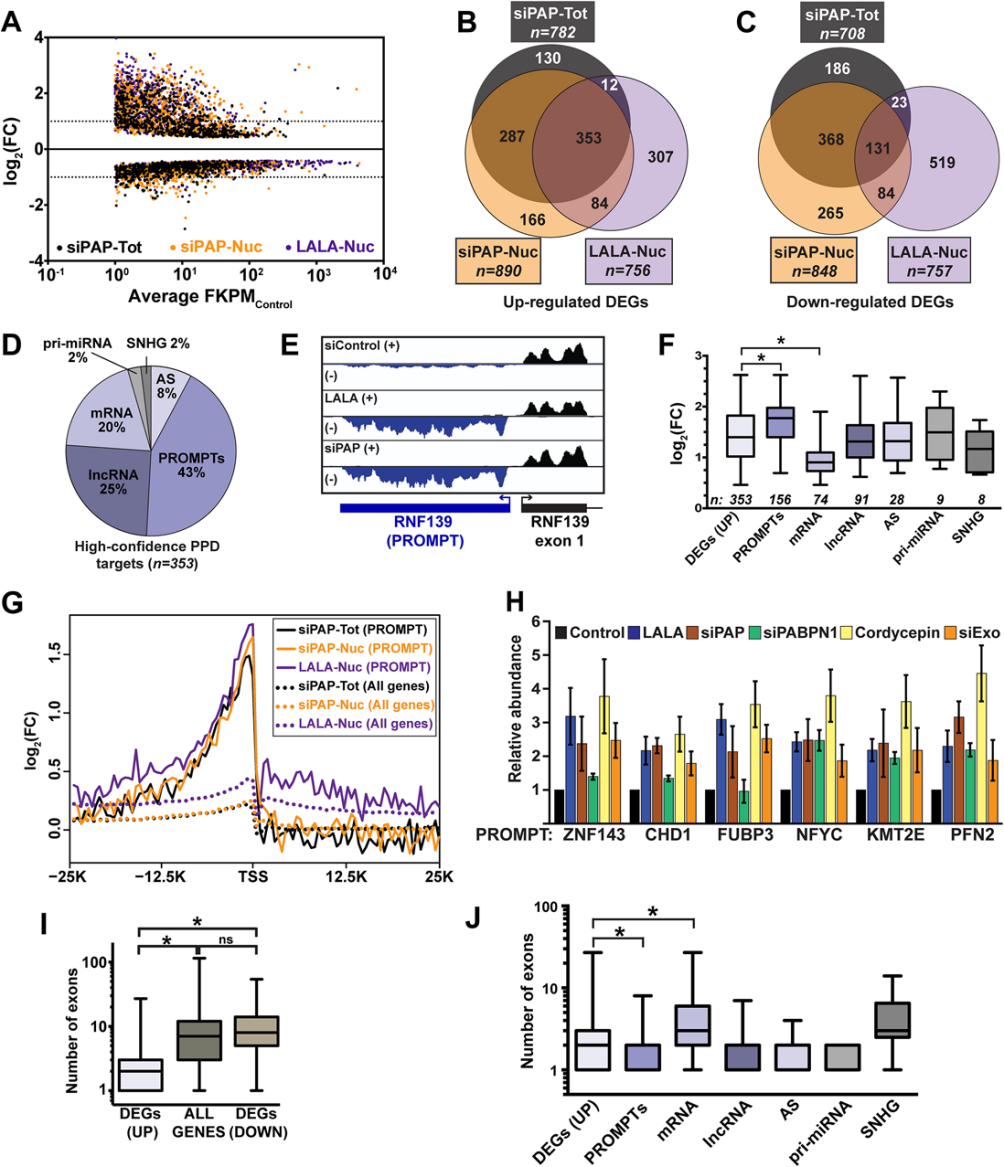
Global analysis of PPD targets. *(A)* Scatter plot of DEGs from each of the three datasets tested. The log_2_ fold change (FC) is relative to the untreated total or nuclear RNA as appropriate. The x-axis is an average FPKM of the control samples for the two biological replicates. *(B)* Venn diagram of the upregulated DEGs identified in each of the three samples. *(C)* Venn diagram of the downregulated DEGs identified in each of the three samples. *(D)* Pie chart of the annotations assigned to the 353 high-confidence upregulated DEGs. *(E)* Strand-specific sequence traces from the RNF139 locus. The plus strand is in black; the minus strand is in blue. *(F)* Box-and-whiskers graph of the fold change of the siPAP total samples for each of the high-confidence target categories. For all box-and-whisker plots, the box corresponds to the 25^th^ through the 75^th^ percentile, the horizontal line is the median and the whiskers represent the upper and lower 25 percent. In this graph, asterisks indicate a p-value <0.0001 (Mann-Whitney test). Thirteen RNAs were categorized into two groups. *(G)* Composite RNA profiles comparing the fold changes from the high-confidence PROMPTs (solid lines) or the entire genome (dotted lines). The data are from one biological replicate; the other replicate is shown in S1B Fig. *(H)* Bar graph of results from qRT-PCR of six PROMPTs under five PPD inactivation conditions as listed. The values are averages and the error bars are standard deviation (*n = 3*). *(I)* Box-and-whisker plots of the number of exons for the up- or downregulated DEGs or all expressed genes. Expressed genes were defined as those with FPKM>1 (*n = 13044*; asterisk, p-value <0.0001; Mann-Whitney test; “ns”, not significant). *(J)* Box-and-whiskers plot of the number of exons for each category of high-confidence targets. (asterisk, p-value <0.0001; Mann-Whitney test).

We identified 1339 differentially expressed genes (DEGs) with increased (upregulated) and 1576 DEGs with decreased (downregulated) levels in at least one PPD inactivation condition (Fig 1A)(S1 Table). We defined high-confidence PPD targets to be those DEGs upregulated in all three datasets (Fig 1B and 1C). Interestingly, 39% (138/353) of the high-confidence transcripts mapped to unannotated loci in the reference genome, while only one of the 131 overlapping downregulated genes (0.8%) was unannotated. We visually inspected the sequence traces of all high-confidence transcripts and categorized them as mRNAs or one of several classes of ncRNA: promoter upstream transcripts (PROMPTs, also known as TSSa-RNAs)[21,22], antisense RNAs (AS), primary miRNA (pri-miRNA), ncSNHG, or lncRNA (S2 Table). Most of the RNAs were ncRNAs (80%, Fig 1D). We additionally performed an independent bioinformatic analysis utilizing a dataset including nearly 14,000 known and novel annotated lncRNAs (GENCODE)(see Materials and Methods). For this analysis, we observed 1178 upregulated lncRNA DEGs in at least one PPD inactivation condition and 408 of these were identified in all three data sets (S1C–S1E Fig and S3 Table). Thus, a considerable number of noncoding polyadenylated nuclear RNAs accumulate upon LALA overexpression and PAP knockdown, suggesting that these transcripts are PPD substrates.

Eukaryotic promoters produce bidirectional transcripts, but generally only one direction produces a stable RNA [22–25]. With respect to number and fold change, PROMPTs were the most responsive class of PPD targets (Fig 1D–1F). Importantly, composite RNA profiles confirmed that our visual assignment of PROMPT was accurate (Fig 1G and S1B Fig). Interestingly, we observed a small peak upstream of the transcription start site (TSS) when the entire genome was used for the composite (dotted lines), suggesting an effect beyond our high-confidence targets (solid lines). We validated the response of six PROMPTs to several PPD inactivation strategies (Fig 1H). In addition to LALA expression and PAP knockdown, we knocked down PABPN1 (siPABPN1), or co-depleted the two catalytic components of the exosome, DIS3 and RRP6 (siExo)(S1A Fig). We also inhibited poly(A) tail extension using cordycepin, an adenosine analog that acts as a chain terminator for poly(A) polymerase due to the absence of a 3´ hydroxyl group. As expected, the levels of the PROMPTs increased upon PPD inactivation, but in some cases PABPN1 knockdown did not have an effect. This is likely due to a general impairment of transcription upon PABPN1 depletion (see below).

We previously reported that an intronless β-globin reporter RNA is degraded by PPD, but its spliced counterpart is stable [5]. Therefore, we tested whether there was a correlation between number of exons and PPD susceptibility. We found that upregulated genes had significantly fewer exons (median 2) than genes from the reference list (median 7), or downregulated DEGs (median 8)(Fig 1I). Noncoding RNAs tend to have fewer exons than protein-coding mRNAs, so our results could be explained by the high proportion of ncRNAs in our dataset, rather than a direct consequence of reduced number of exons. However, even mRNA targets had significantly fewer exons than genes from the reference list (median 3 vs median of 7, p<0.0001). Moreover, the fold changes upon PPD inactivation inversely correlated with the number of exons (S2 Fig). We conclude that PPD substrates have on average fewer exons than transcripts that are not targeted by PPD. Nonetheless, a number of decay targets are spliced, demonstrating that a single splicing event is not always sufficient to confer resistance to PPD.

While mRNA targets had significantly fewer exons than the reference genes, the mRNA targets had more exons than other PPD target categories except ncSNHGs (Fig 1J). Interestingly, mRNAs also had a significantly lower fold-change upon PPD inactivation (Fig 1F) and mRNAs were expressed at higher basal levels than all other classes except ncSNHGs (S1F and S1G Fig). These data suggest within the cellular pool of the specific PPD-susceptible mRNAs, a subset is exported and thereby escapes PPD. As a result, the mRNAs are less affected by PPD inactivation than PROMPTs, which are presumably not exported.

### PPD degrades a subset of noncoding snoRNA host genes

**Fig 2:**
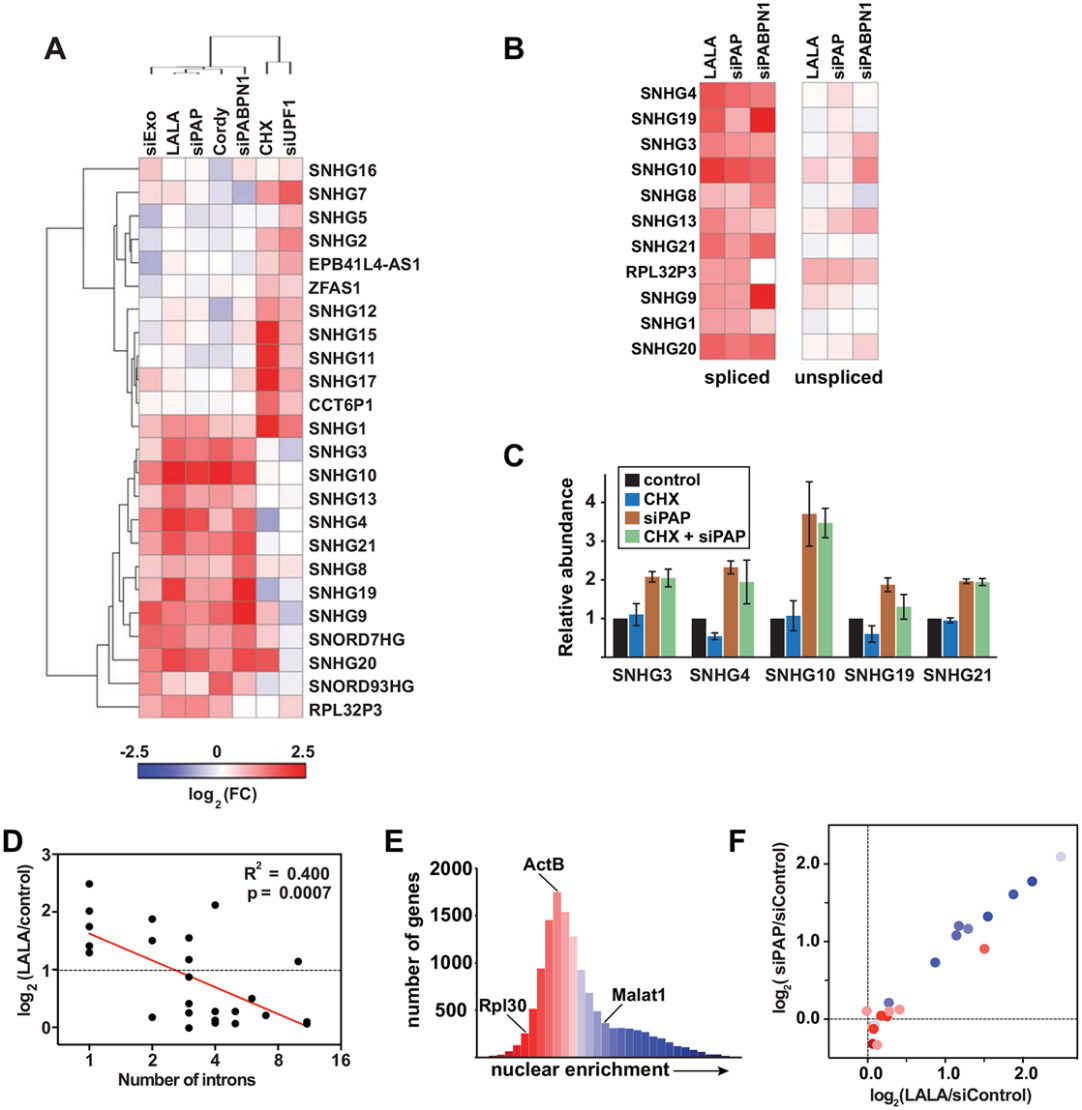
NcSNHGs are degraded by PPD or NMD. *(A)* Heat map showing the changes in spliced ncSNHG levels following RRP6 and DIS3 knockdown (“siExo”), LALA expression, PAP knockdown, cordycepin treatment, PABPN1 knockdown, cycloheximide (CHX) treatment, or UPF1 knockdown. Log_2_ fold change (FC) values were determined by qRT-PCR *(n = 3)*. 7SK RNA was used as a loading control for the cycloheximide experiment, while β-actin or GAPDH was used for all other samples. *(B)* Same as in (A), but the relative changes in spliced (left) and unspliced (right) transcripts are shown. The left panel is reproduced from (A). *(C)* Bar graphs of qRT-PCR data comparing the average relative levels of six ncSNHGs following cycloheximide, siPAP or both treatments. The error bars are standard deviation (*n = 3*). *(D)* Correlation between intron number and the fold change in transcript levels following LALA expression. Expression values are derived from the experiments in (A); the red line is a linear regression. *(E)* Nuclear enrichment scores calculated from each expressed gene (FPKM>0.5, *n = 13*,*114*) were placed into 32 bins and color-coded from red to blue. *(F)* Each ncSNHG was plotted by the average log_2_(FC) values from (A) and color-coded by its NES as determined in (F).

Most mammalian snoRNAs are excised from introns, but the host genes can produce either coding or noncoding RNAs [26]. We identified several ncSNHGs in our RNA-seq analysis and additional ncSNHGs were upregulated that did not meet our stringent cutoffs. In order to obtain a more complete list of ncSNHG PPD targets, we performed qRT-PCR on 24 ncSNHGs expressed in our cell line following inactivation of PPD by several independent methods. In addition, we inactivated NMD by cycloheximide treatment, which indirectly inhibits NMD by inhibiting translation, or by knocking down the NMD factor UPF1 (S1A Fig). Strikingly, we observed largely non-overlapping clusters of ncSNHGs targeted by NMD or PPD (Fig 2A). No upregulation was observed when we used primers that detect the intron-containing transcripts (Fig 2B), so PPD targets the spliced product. We next examined the effects of inactivating both pathways simultaneously. We reasoned that ncSNHGs that evade PPD in the nucleus may be exported and degraded by NMD in the cytoplasm. However, simultaneous PAP knockdown and cycloheximide treatment did not lead to additive accumulation of PPD targets (Fig 2C), suggesting that NMD does not simply degrade ncSNHGs that escape PPD. Instead, each ncSNHG is targeted by a specific pathway.

Consistent with our observation that the number of exons inversely correlates with PPD susceptibility, intron-poor ncSNHGs were more likely to be targeted by PPD (Fig 2D). Because NMD and PPD function in the cytoplasm and nucleus, respectively, and splicing promotes mRNA export [27], we reasoned that differences in ncSNHG localization may contribute to PPD-sensitivity. To test this hypothesis, we calculated a nuclear enrichment score (NES) by dividing the fragments per kilobase of exon per million reads mapped (FPKM) in the nuclear dataset by the FPKM value in the total dataset for each expressed gene. Plotting the NESs confirmed that the nuclear lncRNA MALAT1 had a high NES (blue), while ACTB and RPL30 mRNAs received lower scores (red)(Fig 2E). Next, we compared the NES to the fold changes observed upon PPD inactivation and found that PPD targets were typically more nuclear, while non-PPD targets were more cytoplasmic (Fig 2F). Thus, the differences in nuclear retention and number of exons influence susceptibility to PPD. The simplest interpretation of these results is that fewer splicing events lead to less efficient nuclear export, which in turn increases PPD-susceptibility.

### RNAs with retained introns are subject to PPD

**Fig 3:**
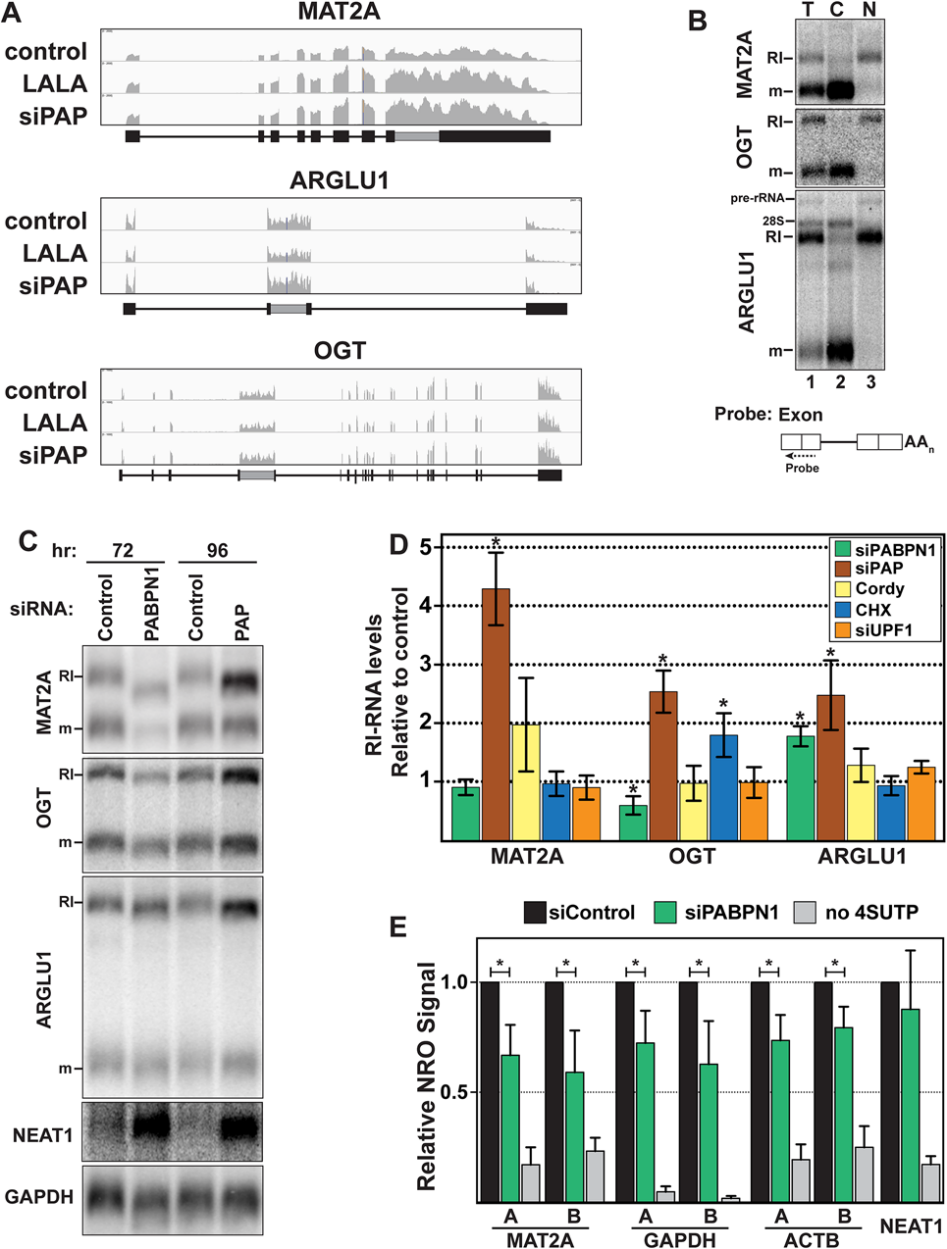
RI-RNAs are subject to PPD. *(A)* Nuclear sequence traces of three RI-RNAs. The RI is shown as a gray box in the gene diagrams. *(B)* Northern blot using total (T), cytoplasmic (C), or nuclear (N) fractions and exon probes that hybridize to both RI and mRNA isoforms. The ARGLU1 probe cross-hybridizes with 28S rRNA; pre-rRNA and rRNA control for fractionation *(C)* Northern blot of specific RNAs from cells treated with siControl, siPABPN1, or siPAP. *(D)* Quantification of the RI isoforms from northern blots (siPABPN1, siPAP, CHX), or qRT-PCR (siUPF1, cordycepin). Each value is normalized to GAPDH or ACTB and expressed relative to the matched control. Error bars are standard deviation from the mean (asterisk, p-value <0.05; unpaired Students t-test; *n = 3*). *(E)* NRO assays using cells treated with control or PABPN1 siRNAs. All values are relative to the control. The no 4SUTP is a negative control in which UTP was substituted for 4SUTP. Two amplicons (labeled “A” and “B”) were used for each gene except NEAT1. Error bars are standard deviation from the mean (asterisk, p-value <0.01; unpaired Students t-test; *n = 4*).

**Fig 4:**
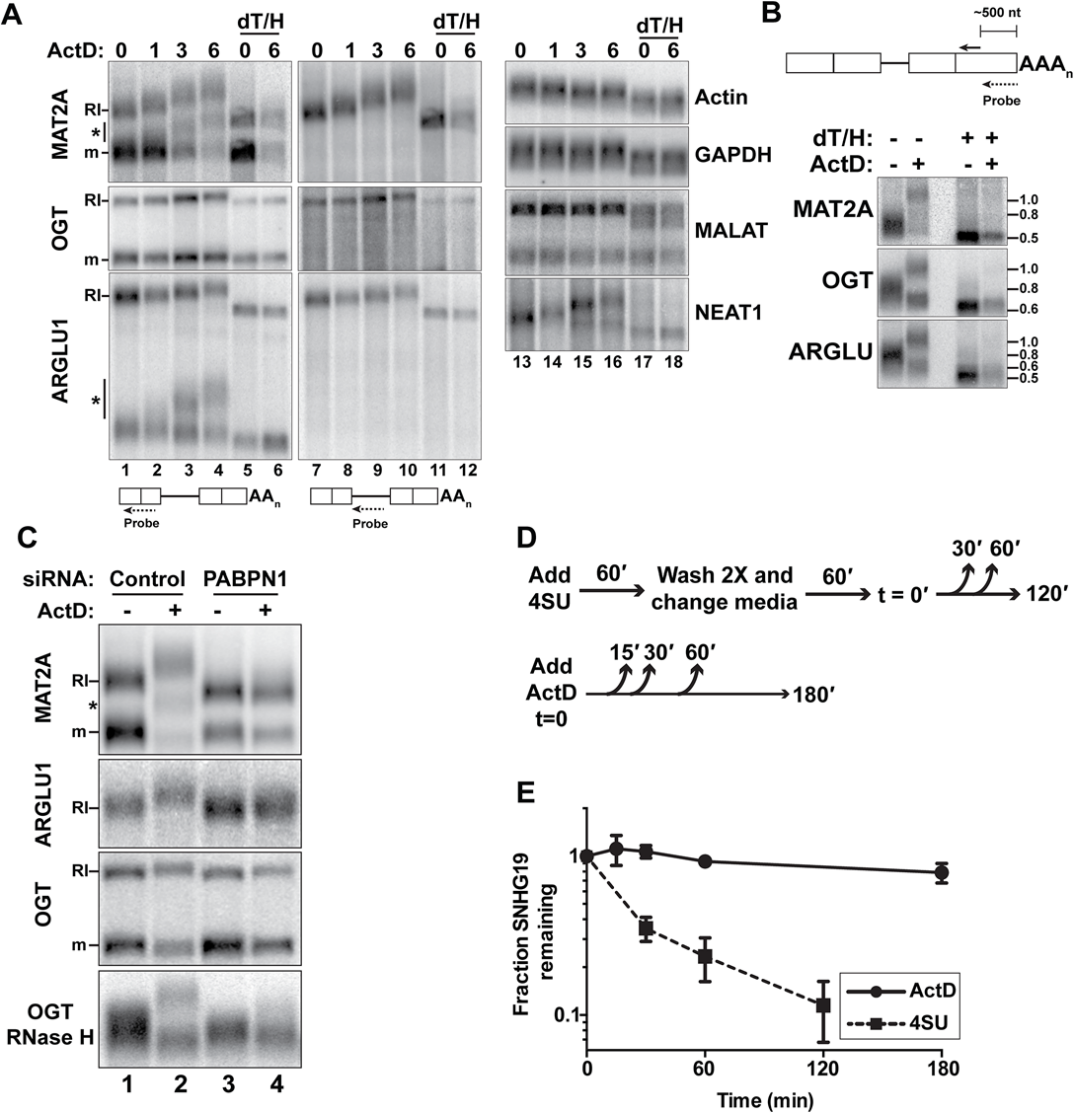
RI-RNAs are hyperadenylated upon ActD treatment. *(A)* Northern blot of RNAs from cells treated with ActD and deadenylated with RNase H and oligo(dT) as indicated. The probes hybridized to exons (lanes 1–6, 13–18) or the RI (lanes 7–12). The asterisk marks RNAs that are fully spliced but hyperadenylated. *(B)* RNAs were cleaved ~500 nt from their poly(A) addition site and examined by northern blot with a probe to the 3´ cleavage product. ActD treatment was for 6 hrs. RNA markers (kb) are shown on the right. *(C)* Northern blots for specific transcripts using RNA from cells transfected with control or PABPN1 siRNAs +/- 6-hr ActD treatment. The bottom panel is an RNase H assay as in (B). *(D)* Scheme of the 4SU pulse-chase and ActD time courses. For the 4SU experiments, cells were washed and grown in label-free media for an additional hour prior to beginning the time course. This step was necessary to allow unincorporated 4SU in the cell to be depleted. *(E)* Decay profiles of SNHG19 as determined by 4SU or ActD.

MAT2A is a high-confidence PPD target and inspection of its sequence traces revealed retention of the 3´-most intron (Fig 3A). Recent studies have established that intron retention is significantly more common in mammals than previously appreciated [28–31]. Retained intron-containing RNAs (RI-RNAs) can be degraded by NMD, but most are degraded in the nucleus by an unknown pathway [28,29,31]. We tested whether PPD affects RI-RNA decay more generally by examining MAT2A and two other RI-RNAs, OGT and ARGLU1. Each gene produced highly expressed nuclear RI-RNAs and fully spliced cytoplasmic mRNAs (Fig 3A and 3B). The presence of the retained intron is verified below (Fig 4A).

Neither ARGLU1 nor OGT was identified as a high-confidence target, but ARGLU1 was upregulated in the siPAP-total and siPAP-nuclear datasets. Similarly, cordycepin treatment increased MAT2A-RI levels ~2-fold, but this effect did not reach statistical significance (p = 0.10) and cordycepin did not affect OGT-RI or ARGLU1-RI levels (Fig 3D). While these data suggest little PPD sensitivity, none of the RI-RNAs responded to UPF1 depletion and only OGT-RI increased in response to cycloheximide, consistent with previous reports that NMD is not the general mode of decay for these RNAs [28,29,31]. To further probe a potential role of PPD in RI-RNA decay, we tested whether timing of the knockdown experiments influenced our results. When we increased siPAP treatment from three to four days, we observed statistically significant upregulation of MAT2A-RI (4.2-fold), OGT-RI (2.5-fold), and ARGLU1-RI (2.5-fold) supporting the conclusion that PPD targets RI-containing RNAs (Fig 3C and 3D).

PABPN1 knockdown increased ARGLU1-RI levels ~1.8-fold, but neither MAT2A-RI nor OGT-RI increased (Fig 3C and 3D). Unlike siPAP treatments, extended knockdown of PABPN1 did not increase RI-RNAs. Moreover, the cell morphology was generally worse for PABPN1 knockdowns compared to PAP knockdowns suggesting greater toxicity. Therefore, we hypothesized that decreases in transcription prevent accumulation of RI-RNAs upon PABPN1 depletion. To test this idea, we performed nuclear run-on (NRO) assays using the modified nucleotide, 4-thiouridine triphosphate (4SUTP), to detect nascent transcripts. We observed a general decrease in Pol II density on several genes after PABPN1 knockdown (Fig 3E). We conclude that steady-state levels of some PPD targets do not increase upon PABPN1 knockdown due to concomitant decreases in RNA synthesis rates. Importantly, we detected no change in transcription upon PAP knockdown (S3C Fig), consistent with our observation that RI-RNAs accumulate after PAP knockdown. We further corroborated the NRO results by examining nascent transcripts from live cells using a metabolic labeling protocol (S3E Fig). These results support a role for PPD in degradation of nuclear RI-RNAs but suggest that the relative rates of transcription and decay of RI-RNAs may differ from the more robustly upregulated ncRNAs such as PROMPTs. We also examined the mRNA isoform of MAT2A, OGT, or ARGLU1, and observed no general trends (S3B Fig). We suggest this is due to distinct half-lives, translation efficiencies, and/or the precursor-product relationship between a specific RI transcript and its cognate mRNA.

### RNAs with retained introns are hyperadenylated and stabilized following general transcription inhibition

Initially, we attempted to examine MAT2A-RI stability by treating cells with the general transcription inhibitor actinomycin D (ActD). As expected, the mRNA degraded over time (Fig 4A). Surprisingly, the MAT2A-RI isoform was robustly hyperadenylated upon ActD treatment and the transcript persisted. We verified that this transcript corresponded to the MAT2A-RI by stripping and re-probing with a retained-intron specific probe (lanes 7–12). In addition, ARGLU1-RI and the OGT-RI transcripts were stable and hyperadenylated after ActD treatment (Fig 4A). Because these transcripts are longer than MAT2A-RI, the hyperadenylation was not as obvious as for MAT2A. Therefore, we cleaved the transcripts ~500 nt from their 3´ ends using RNase H and a specific targeting DNA oligonucleotide and examined the 3´ fragment prior to and after ActD treatment (Fig 4B). Hyperadenylated and shorter poly(A) tails were readily detected, reflecting the RI and mRNA isoforms, respectively. After ActD treatment, the hyperadenylated tails ranged from ~300–800 nt, while mRNAs were ~50–200 nt (S4 Table). NEAT1, a known ncRNA PPD target [4], was also hyperadenylated after ActD treatment (Fig 4A, lanes 13–18). In contrast, neither β-actin nor GAPDH mRNAs displayed poly(A) tail extension upon ActD treatment (lanes 13–18). Moreover, the nuclear ncRNA MALAT1, which does not have a poly(A) tail [32], was not extended upon ActD treatment.

MAT2A and ARGLU1 RNAs of intermediate lengths were hyperadenylated after ActD treatment (Fig 4A, asterisks). We observed only two bands corresponding to fully spliced and RI-RNAs after RNase H/oligo(dT) treatment, so we conclude that these RNAs are spliced, but still subject to hyperadenylation and nuclear retention. (S3D Fig). We discuss possible mechanisms of production of these RNAs in the Discussion section.

PABPN1 knockdown prevents the hyperadenylation of RI-RNAs after ActD treatment (Fig 4C, compare lanes 2 with 4). PABPN1 depletion also decreased the length of MAT2A-RI in the untreated samples (lanes 1 and 3), but the MAT2A mRNA lengths were largely unaffected. Similar results were observed with PAP knockdown (Fig 3C). Thus, PABPN1 and PAP hyperadenylate MAT2A-RI even in control cells and similar results were observed with ARGLU1-RI and OGT-RI isoforms (Fig 4C). If PABPN1 knockdown released RI-RNAs from the nucleus, the shorter poly(A) tails could be due to cytoplasmic deadenylation. However, the RI-RNAs remained predominantly nuclear upon PABPN1 depletion (S3A Fig). We conclude that RI-containing transcripts have longer poly(A) tails due to PABPN1 and PAP activity, and that this effect is exacerbated following treatment with ActD.

MAT2A-RI is targeted by PPD, but upon ActD treatment the poly(A) tail is extended and the RNA is relatively stable. One interpretation of this finding is that ActD treatment decouples hyperadenylation from decay. To test this with a different PPD target, we compared the half-lives of SNHG19 after ActD treatment with a 4SU metabolic pulse-chase assay that does not require general transcription inhibition (Fig 4D). The apparent half-life of SNHG19 in ActD was >3hr, while the pulse-chase method yielded a <30 min half-life (Fig 4E). These observations show that some PPD targets are stabilized by general transcription inhibition and highlight the potential caveats of using general transcription inhibitors to monitor nuclear RNA half-lives.

### Transcription shut-off induces the hyperadenylation of bulk nuclear RNAs

**Fig 5:**
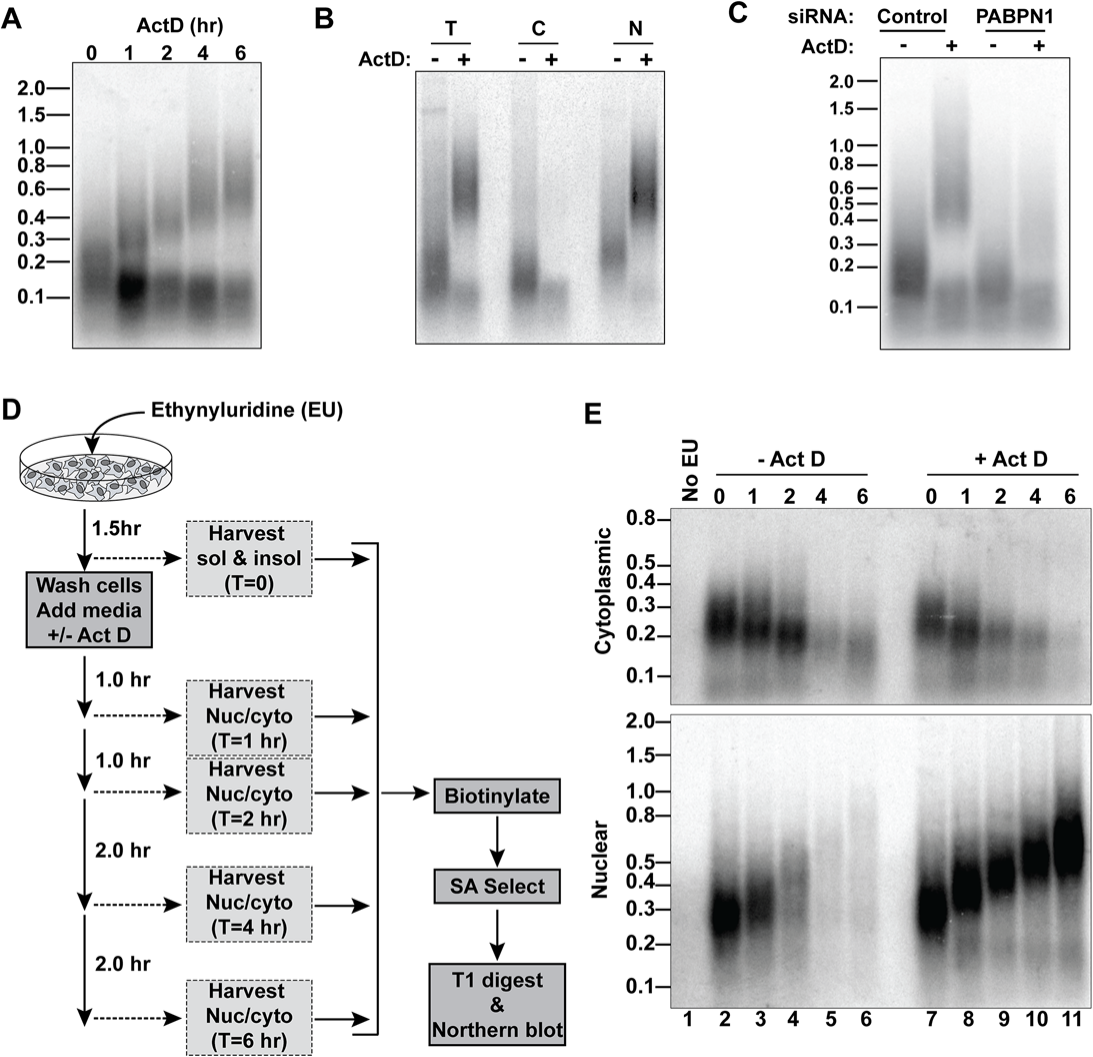
A large fraction of nuclear RNA is hyperadenylated upon ActD treatment. *(A)* Total cellular poly(A) tails were examined by northern blot after ActD treatment. Mobility of molecular weight markers (kb) is shown. *(B)* Bulk poly(A) tails were examined from untreated or ActD treated cells from whole cells (T), cytoplasmic (C) or nuclear (N) fractions. *(C)* Northern blot for bulk poly(A) tails using RNA from cells treated with a control siRNA or siRNAs targeting PABPN1 +/-6-hr ActD treatment. *(D)* Scheme for metabolic labeling approach to examine bulk poly(A) tail dynamics. (E) Results of a metabolic labeling assay examining the bulk soluble and insoluble poly(A) tails in cells +/- ActD treatment. Lane 1 is a negative control from cells without EU treatment.

To explore the generality of the ActD-induced hyperadenylation, we collected RNA from cells treated with ActD over a 6-hr time course and digested them with RNase T1, a G-specific endonuclease, to degrade transcripts but leave poly(A) tails intact. We then detected bulk poly(A) tails by northern blot with an oligo(dT)_40_ probe (Fig 5A). After ActD treatment, one subset of poly(A) tails lengthened, while another population shortened over time. We observed similar effects with 5,6-dichloro-1-β-D-ribofuranosylbenzimidazole (DRB), flavopiridol, and triptolide, which inhibit transcription by mechanisms distinct from ActD (S4A Fig) [33]. Moreover, this hyperadenylation was observed in HeLa cells and primary mouse macrophages, so the effect is neither cell-type nor species-specific (S4B Fig). Admittedly, the fraction of RNAs hyperadenylated is lower than its appearance on the northern blots (Fig 5A) because more oligo(dT)_40_ probes will hybridize to the longer tails to increase the signal, but the hyperadenylated transcript pool nonetheless comprises a large fraction of the total poly(A) RNA.

The two bulk poly(A) pools closely mimicked our observations with RI-RNA and mRNA isoforms. For example, the shorter population was primarily cytoplasmic whereas the hyperadenylated RNAs were nuclear (Fig 5B). Moreover, the poly(A) tails were longer in the nuclear pool even in the absence of ActD and hyperadenylation was diminished in PABPN1-depleted cells (Fig 5C). Next, we used a metabolic pulse-chase assay to examine bulk poly(A) tail dynamics (Fig 5D). As expected, the cytoplasmic poly(A) tails shortened over time and ActD did not appreciably change this pattern (Fig 5E). In the absence of ActD, the nuclear poly(A) tails grew longer but disappeared over time. In contrast, in the presence of ActD, the nuclear poly(A) tails persisted and were continually extended, thereby mirroring the hyperadenylation and lack of nuclear decay observed with specific PPD substrates (Fig 4). We conclude that a large fraction of nuclear polyadenylated RNA is subject to hyperadenylation and stabilization upon general transcription inhibition.

### Role of hyperadenylation in PPD

**Fig 6:**
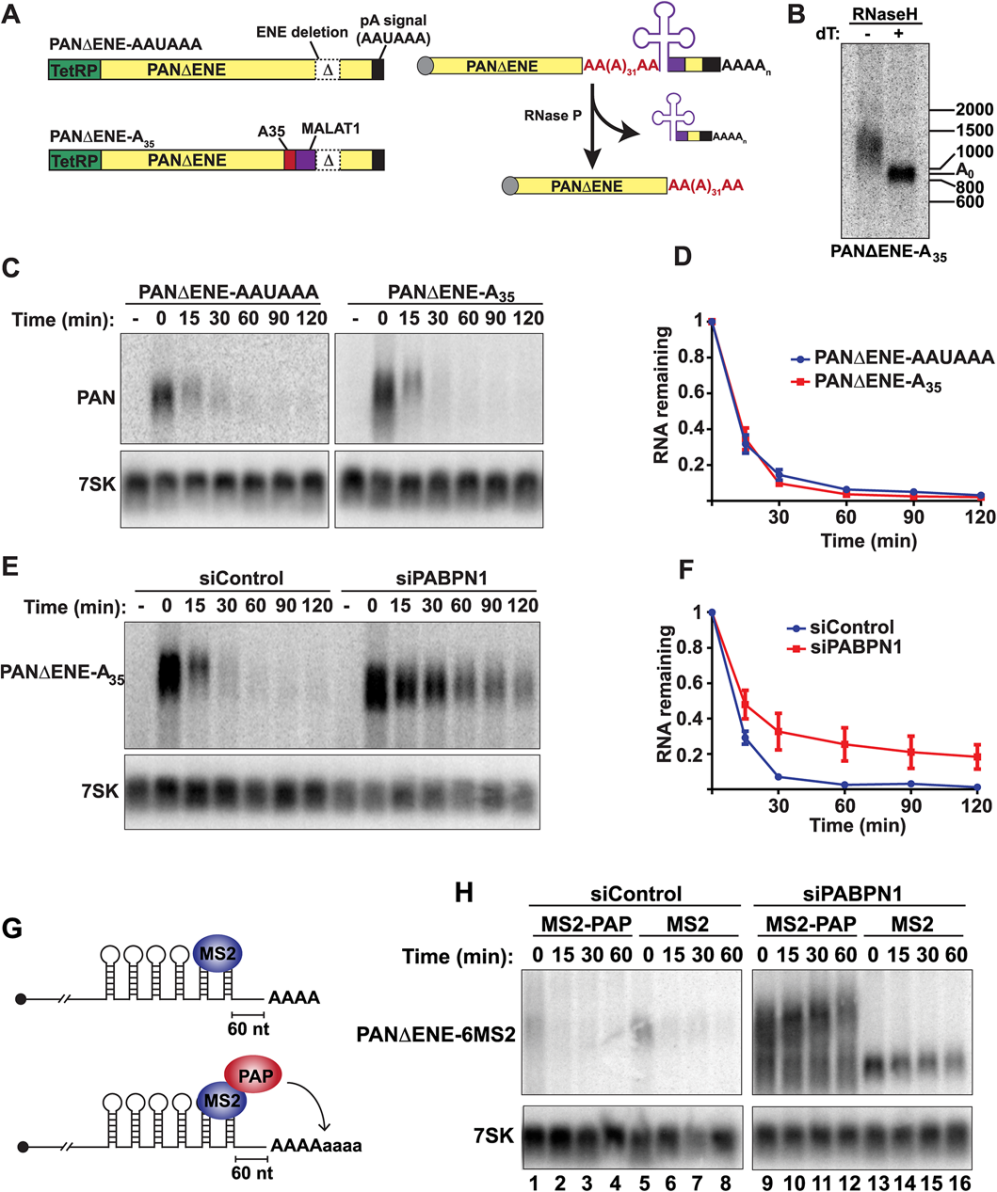
Role of hyperadenylation in PPD. *(A) Left*, Cartoons of the PANΔENE-AAUAAA and PANΔENE-A_35_ plasmids depicting the TetRP (green), PAN RNA sequence (yellow), PAN RNA polyadenylation signals (black), A_35_ stretch (red), MALAT1 3´-end cleavage sequence (purple), and the position of the ENE deletion (Δ); the diagrams are not to scale. *Right*, Scheme of the production of PANΔENE-A_35_ by RNase P cleavage in cells. The color scheme is the same as the DNA diagrams; the cap is shown as a gray circle. The MALAT1 mascRNA sequence is represented by the cloverleaf structure. *(B)* Poly(A) tail length analysis of PANΔENE-A_35_. RNA was harvested and treated with RNase H in the presence or absence of oligo(dT). *(C)* Representative transcription pulse-chase assay with the indicated constructs. The “-” samples were harvested prior to the two-hour transcription pulse. 7SK RNA was used as a loading control. *(D)* Quantification of the pulse-chase assays; error bars show the standard deviation of the mean *(n = 3)*. (*E* and *F*) Representative transcription pulse chase and quantification of PANΔENE-A_35_ following treatment with the indicated siRNAs (*n = 3*) *(G)* Illustration of the PAP-tethering approach. (H) Transcription pulse-chase analysis of TetRP-driven PANΔENE containing six MS2 binding sites. Cells were treated with either control (left) or PABPN1 (right) siRNAs. Cells were co-transfected with PANΔENE-6MS2 and either MS2-PAP or MS2 expression constructs.

PABPN1 and PAPα/γ are components of the 3´-end formation machinery, but whether other components, like CPSF, are involved in PPD is unknown. Even though hyperadenylation occurs after the initial polyadenylation event, CPSF may remain bound to the PAS and influence hyperadenylation or decay. To test this, we took advantage of the unusual processing of the MALAT1 lncRNA. The MALAT1 3´ end is generated by RNase P, which cleaves directly upstream of a tRNA-like element in the RNA [32]. We cloned the tRNA-like element into a TetRP-driven ENE-lacking PAN RNA reporter immediately downstream of a 35-nt A stretch (Fig 6A)(PANΔENE-A_35_). The processing at the MALAT1 cleavage site is efficient, with ~85% of the RNAs being cleaved by RNase P after a 2-hr transcription pulse (S5A Fig). In cells, the A_35_ tail was extended to ~100–500 nt (Fig 6B). Importantly, the cleaved transcript lacks an AAUAAA site, so this extension was independent of CPSF. To examine PANΔENE-A_35_ stability, we used a TetRP-based transcription pulse-chase strategy. After a 2-hr transcription pulse, we monitored stability of PANΔENE-A_35_ and PANΔENE with its natural PAS (PANΔENE-AAUAAA) and observed indistinguishable decay kinetics (Fig 6C and 6D). Moreover, knockdown of PABPN1 (Fig 6E and 6F) or LALA expression (S5B Fig) stabilized PANΔENE-A_35_. Thus, PPD does not strictly require CPSF or a PAS.

PABPN1, but not CPSF, stimulates polyadenylation after the initial processive polyadenylation step by increasing PAP association with RNA [13]. We previously proposed that this in vitro activity reflects the hyperadenylation required for PPD, which is further supported by the demonstration that PPD can occur in a CPSF-independent fashion (Fig 6A–6F). In principle, stimulation of hyperadenylation could be the sole requirement for PABPN1 in PPD. To test this hypothesis, we bypassed the requirement for PABPN1 in hyperadenylation by tethering PAP directly to PANΔENE RNA. We inserted six bacteriophage MS2 coat protein binding sites into PANΔENE upstream of the poly(A) tail, which allows us to tether an MS2-PAP fusion protein to PAN RNA in cells (PANΔENE-6MS2)(Fig 6G). When MS2-binding protein was expressed, PANΔENE-6MS2 was rapidly degraded in control cells (Fig 6H, lanes 5–8), but stabilized upon PABPN1 knockdown (Fig 6H, lanes 13–16). When we co-expressed PANΔENE-6MS2 with MS2-PAP, PANΔENE-6MS2 was rapidly degraded in control cells as expected (Fig 6H, lanes 1–4). Importantly, MS2-PAP was unable to rescue decay after PABPN1 depletion, despite the fact that PANΔENE-6MS2 was hyperadenylated (Fig 6H, lanes 9–12). Therefore, hyperadenylation is not sufficient to stimulate PPD in the absence of PABPN1, suggesting that PABPN1 serves multiple functions in PPD by promoting hyperadenylation and an additional step in RNA decay.

## Discussion

The mechanisms and regulation of nuclear RNA decay remain poorly defined, particularly in mammalian cells. Here we show that several classes of nuclear noncoding RNAs are subject to degradation by PPD including upstream antisense RNAs, ncSNHGs, pri-miRNAs, lncRNAs, and antisense transcripts. Our observations are consistent with global analyses reported by Bachand and colleagues demonstrating that PABPN1 knockdown leads to the stabilization of nuclear lncRNAs [4]. In addition, our RNA-seq and knockdown analyses revealed that specific canonical mRNAs and RI-containing RNAs are PPD targets. By using PAP knockdown and PAP-stimulation deficient PABPN1 mutant LALA as the basis of our RNA-seq experiments, these data confirm that PAP activity is necessary for the degradation of a large collection of nuclear RNAs. Given the parameters used in the RNA-seq analysis, it is likely that our high-stringency dataset is an underestimate of the number of RNAs subject to PPD. For example, a subset of ncSNHGs and the RI-RNAs were confirmed to be PPD substrates by qRT-PCR (Fig 2A) and northern blot (Fig 3C and 3D) even though these RNAs were not identified in our RNA-seq study. Based on these global and mechanistic studies we conclude that PPD is a major RNA decay pathway for nuclear polyadenylated transcripts.

The PROMPTs were the most PPD sensitive transcripts based on their fold changes upon PPD inactivation (Fig 1F) and their overrepresentation among DEGs (Fig 1D). Pervasive transcription from bidirectional promoter firing is a common feature in eukaryotes [1,22,23,25,34,35]. In *S*. *cerevisiae*, the resulting divergent transcripts are terminated by the Nrd1-Nab3-Sen1 (NNS) pathway due to an over-representation of binding sites for the Nrd1p and Nab3p proteins upstream of yeast promoters [36,37]. The multisubunit Trf4-Air2-Mtr4 polyadenylation (TRAMP) complex then targets the NNS-terminated fragments to the nuclear exosome [38–40]. In contrast, promoter directionality in mammalian cells is achieved by an enrichment in canonical PASs in the upstream antisense direction and depletion of U1 snRNP binding sites [41,42]. At least some PROMPTs are terminated by the combined actions of the canonical cleavage and polyadenylation machinery, the cap-binding complex and its associated protein ARS2 [41–44]. After termination, the trimeric NEXT complex targets PROMPTS for decay by the exosome [24,43,45,46]. In addition, bidirectional transcripts can be terminated and degraded by co-transcriptional decapping and 5´→3´ decay by Xrn2 [47]. Three studies, including this one, report that specific PROMPTs are degraded in a PABPN1-dependent fashion [4,48]. Visual inspection of the sequence traces of previously published NEXT-sensitive PROMPTS is ambiguous regarding their susceptibility to PPD (S6A Fig) [4], suggesting that specific PROMPTs are targeted by distinct nuclear decay pathways. Further experimentation is required to determine whether the PPD, Xrn2 and NEXT pathways target independent subsets of upstream antisense transcripts, or are largely redundant pathways for bidirectional transcript degradation.

U1 snRNP is a core component of the spliceosome that recognizes 5´ splice sites, but it also suppresses the use of premature PASs [49,50]. This latter function contributes to promoter directionality in that U1 snRNP binding sites are depleted in upstream antisense regions and overrepresented in coding regions [41,42]. As a result, antisense transcription normally produces shorter, unspliced transcripts, whereas coding genes produce longer spliced pre-mRNAs. Interestingly, five of our high-confidence PPD substrates classified as mRNAs had increased sequence coverage at the 5´ end of the genes (APOLD1, MTHFD2L, AGBL3, TEX22, and FAM120C)(S6B Fig). We speculate that these transcripts result from a failure of U1 snRNP to protect from premature PAS usage. The resulting RNAs resemble promoter antisense RNAs and are therefore subject to degradation by PPD. This speculation is supported by a recent global analysis demonstrating that PABPN1 depletion increased the levels of similar sense proximal RNAs [48].

We previously demonstrated that an intronless β-globin reporter is rapidly degraded by PPD, but insertion of a single intron into that reporter is sufficient to protect the resulting mRNA from PPD [5]. Consistent with this idea, 174/353 (49%) of the high-confidence RNAs identified are single-exon RNAs (S2 Fig). The simplest explanation for this observation is that splicing promotes the formation of an export-competent mRNP leading to export and escape from PPD [27]. However, a single splicing event is not always sufficient to promote escape from PPD. By definition, all PPD-targeted ncSNHGs are spliced at least once (Fig 2) and only 5/74 PPD-sensitive mRNAs are single exon genes (S2 Table). Because ncSNHGs targeted by PPD had higher nuclear enrichment (Fig 2), we conclude that PPD susceptibility stems from nuclear retention of the spliced transcript. This could be due to nuclear retention signals in the exons or due to variations in recruitment of splicing-dependent export factors.

We also found that RI-RNAs are subject to PPD (Figs 3 and 4). Recent studies point out the importance of intron retention in mammalian cells [28–31]. The efficiency of splicing of these retained (“detained” in [31]) introns can be modulated by developmental or environmental cues supporting an essential role for these RNAs in posttranscriptional gene regulation. These previous studies showed that a subset of RI-RNAs is degraded by NMD while others are retained in the nucleus and degraded by a previously unknown nuclear RNA decay pathway. Our data now show that that nuclear retained RI-RNAs are subject to PPD. Thus, there is a parallel between RI-RNAs and ncSNHGs in that both produce spliced RNAs that are either exported and subject to NMD or retained in the nucleus and subject to PPD. Importantly, the RI-RNAs are not strongly upregulated by PPD inactivation. We had to increase the lengths of time for PAP knockdown to observe increases in ARGLU1 and OGT and cordycepin treatment had no effect on their abundance (Fig 3D). This may be due to the biology of the RI-RNAs. For example, if they serve as precursors to pre-mRNAs as proposed [31,51], the half-lives of these RNAs may be longer than the nonfunctional ncSNHGs or PROMPTs. Thus cells may regulate PPD to control the accumulation of RI-RNAs. Given the widespread use of intron retention in mammals, PPD regulation may have important consequences for gene expression. Interestingly, PABPN1 was recently shown to autoregulate its mRNA levels by intron retention [52].

Testing the half-lives of the nuclear RNAs identified herein is complicated by the unusual behavior of nuclear RNAs upon general transcription inhibition (Figs 4 and 5). We do not understand how transcription inhibition leads to the accumulation of hyperadenylated nuclear RNAs, but the simplest explanation for this striking phenomenology is that PABPN1-dependent hyperadenylation occurs, but is uncoupled from the decay step of PPD. We stress that this is not the result of a specific transcription inhibitor or concentration as four different transcription inhibitors, which utilize at least three distinct mechanisms of transcription inhibition yielded a similar result (S4 Fig). Interestingly, we observed that a portion of completely spliced MAT2A and ARGLU1 RNAs was hyperadenylated after ActD treatment (Fig 4A and S3D Fig). Because there is little fully spliced RNA in the nuclear fraction prior to ActD treatment (S3D Fig), it seems likely that the retained intron is posttranscriptionally spliced. However, this splicing is not sufficient to release the RNA for export, at least in the presence of ActD. Perhaps transcription inhibitors indirectly produce a general block in mRNA export. Alternatively, the RI-RNAs may be fated for the discard pathway, so they are subject to nuclear retention and PPD even after splicing. Another explanation is that the RI-RNAs are normally degraded, but ActD-induced stabilization (Figs 4 and 5) allows sufficient time for the RNAs to be fully spliced. Given the prevalence of intron retention in mammals, the interrelationships between PPD, splicing, and transcription warrant deeper investigation.

In yeast, the TRAMP complex component Trf4, a noncanonical poly(A) polymerase, marks nuclear RNAs for decay by the exosome. While Trf4 is essential for decay, its polyadenylation activity is not necessary [53–55]. In contrast, our studies are consistent with the conclusion that hyperadenylation of PPD targets is linked to their decay. Transcripts that are upregulated following PABPN1-depletion are also increased following depletion of PAP or expression of a polyadenylation defective PABPN1 allele (Figs 1 and 2). Three lines of evidence suggest that distributive rather than processive polyadenylation is the primary driver of decay. First, CPSF is necessary for processive polyadenylation in vitro so the CPSF-independent PANΔENE-A_35_ is unlikely to undergo processive polyadenylation. Nevertheless, PANΔENE-A_35_ was degraded by PPD (Fig 6), suggesting that processive polyadenylation is dispensable for decay. Second, a distributive process should be more sensitive to relative concentrations of PPD factors in the cell because of the requirement for re-binding after dissociation. Indeed, our siPAP knockdowns decrease PAP levels such that hyperadenylation is affected, but there appears to be little effect on the initial polyadenylation reaction [5]. Third, upon transcription inhibition, poly(A) tails gradually increased in length as a group over several hours, consistent with PAP disassociating and re-associating with transcripts stochastically (Figs 4 and 5). In contrast, processive polyadenylation that forms the initial poly(A) tail occurs rapidly in vitro and in cells with ~200–250 nucleotides being added in less than one minute [56,57]. Interestingly, even though PABPN1 stimulates CPSF-independent distributive hyperadenylation, hyperadenylation was not sufficient to rescue PPD sensitivity in the absence of PABPN1 (Fig 6H). Thus, PABPN1 likely plays multiple roles in PPD. In fact, Pab2 and PABPN1 co-immunoprecipitate with the exosome [4,58], suggesting PABPN1 may directly recruit the exosome. Alternatively, PABPN1 may compete with poly(A) binding proteins that stabilize RNAs. Thus, upon PABPN1 depletion, these proteins preferentially associate to increase RNA half-lives [59,60].

In summary, our data show that PPD modulates the levels of functional lncRNAs and mRNAs as well as presumably nonfunctional PROMPTs and the spliced byproducts of snoRNA and pri-miRNA processing. We conclude that PPD is an important nuclear RNA decay pathway that lies at the interface of transcription, splicing, 3´-end formation and mRNA export.

## Materials and Methods

### RNA-seq and identification of DEGs

RNA-seq and sequencing was performed at the McDermott Center Next Generation Sequencing Core and Bioinformatics Core. Libraries were prepared using the TruSeq Stranded mRNA preparation kit and run on an Illumina HiSeq 2500 (paired-end 100 bp reads). The reads were mapped, aligned and assembled using TopHat2 and Cufflinks2.2 [61,62]. Transcriptome assembly was guided by iGenomes (hg19, UCSC build) and GENCODE (release 19) annotation files. Differential gene expression was analyzed by Cuffdiff using the iGenomes annotations and EdgeR was employed to determine differential expression of the 13,853 known and novel lncRNAs in the GENCODE annotation [63]. Integrative genomics viewer (IGV) was used to visualize sequence coverage and generate figures [64]. DEGs were identified from the Cuffdiff output by removing those transcripts with an FPKM of <1 in the treatment sample and the remaining transcripts with p-value <0.05 and a false discovery rate (FDR) less than 5% were defined as DEGs (S1 Table). DEGs in the EdgeR data were defined as those with log(counts per million) >3.5 and an FDR <5% (S3 Table). Heat maps were generated using the GENE-E software (http://www.broadinstitute.org/cancer/software/GENE-E/index.html).

We categorized each of the 353 high-confidence upregulated DEGs by visual assessment of IGV traces (S2 Table). Any DEG found upstream and antisense to an annotated gene was defined as a PROMPT. Antisense orientation was confirmed in IGV using strand-specific bigWig files generated by HOMER [65]. AS transcripts, on the other hand, were those with considerable overlap within an annotated gene. Pri-miRNA and ncSNHG transcripts were inferred by the presence of an overlapping miRNA/snoRNA or corresponded to annotated genes. We assigned the category lncRNA to any transcript that was from an annotated lncRNA gene or from an unannotated genomic region that did not fall into any of the other categories.

### Plasmids, transfections and TetRP pulse-chase assays

All plasmids were constructed using standard molecular biology techniques. The details of the construction are given in the Expanded View. Transfections and TetRP pulse-chase assays were performed as previously described [5].

### Detection of newly made bulk poly(A) RNAs

Detection of newly made bulk poly(A) tails was performed essentially as previously described [66,67].

### Northern blotting

Bulk poly(A) tails were detected on 1.8% agarose-formaldehyde gel, and detected with a dT_40_ probe end-labeled with T4 polynucleotide kinase. Northern blots for specific transcripts were performed using standard techniques with RNA probes. Stripping and re-probing of the membranes were performed as previously described [5]. The RNA probes were generated from PCR products with a T7 RNA polymerase promoter; primers are listed in S5 Table. For some northern blots, 35–80 mg of total RNA were selected on oligo(dT) cellulose to enrich for polyadenylated RNAs prior to gel electrophoresis. In addition, we degraded residual rRNA after oligo(dT)-cellulose selection with Terminator exonuclease (EpiCentre).

### Fractionation

To collect cytoplasmic RNA, cells were resuspended in Buffer I (0.32 M sucrose, 3mM CaCl_2_, 2 mM MgCl_2_, 0.1 mM EDTA, 10 mM Tris-HCl (pH 8.0), 1 mM DTT, 0.04 U/ml RNase Inhibitor, 0.5% Triton X-100), incubated on ice for 5 min, centrifuged at 500 x g for 3 min at 4°. RNA in the supernatant was extracted using TriReagent (Molecular Research Center) followed by an additional phenol-chloroform extraction. The pellet was then washed in Buffer I with 150 mM NaCl and once again centrifuged at 500 x g for 3 min at 4°. The resulting supernatant was discarded. The RNA from the remaining pellet was then extracted in TriReagent. We note that in cases in which we analyzed RNA from the wash step, we observed both long and short poly(A) tails; whether this is due to cross contamination of cellular compartments and/or is due to a distinct biological fraction is unclear. This fractionation procedure results in the loss of Triton X100-soluble nuclear material, but it enriches for chromatin and nuclear speckle-associated RNAs [18–20].

### Quantitative RT-PCR

RNA was harvested using TriReagent according to the manufacturer’s protocol. Following extraction, RNA was treated with RQ1 DNase (Promega). Random hexamers were used to prime cDNA synthesis with MuLV reverse transcriptase (NEB). Real-time reactions used iTaq Universal SYBR Green Supermix (Biorad).

### Biotinylation and streptavidin selection

Biotinylation reactions were carried out in a 200μL mixture consisting of 40μg RNA, 20mM NaOAc (pH 5.2), 1mM EDTA, 0.1% SDS, 0.2mg/mL Biotin-HPDP (Pierce), and 50% N,N-dimethylformamide (DMF) for 3 hours at 25°C. Unconjugated biotin-HPDP was removed with three chloroform extractions. After extraction of the aqueous phase, 20μL (10% v/v) of 10M NH_4_OAc was added to each tube, and the RNA was precipitated in 70% ethanol.

Streptavidin selection was carried out using magnetic Streptavidin T1 beads (Invitrogen). Prior to use, the 20 μl bead slurry was washed three times in a 0.1X MPG solution (1X MPG was 1M NaCl, 10mM EDTA, and 100mM Tris 7.5) supplemented with 0.1% igepal. After the final wash, the beads were resuspended in a 1mL solution consisting of 0.1X MPG supplemented with 0.1% igepal, 0.1μg/μL poly(A) (Sigma-Aldrich), 0.1μg/μL ssDNA, 0.1 μg/μL cRNA, and 0.1% SDS, and blocked for one hour. RNA was precipitated, resuspended in a volume of 63μL water, and denatured at 65°C for 5 minutes. Next, RNA was incubated together with beads for one hour while nutating at room temperature. Beads were sequentially washed in: 0.1X MPG, 0.1X MPG at 55°C, 0.1X MPG, 1X MPG, 1X MPG, 0.1X MPG, 1X MPG without NaCl, 0.1X MPG. With the exception of the 55°C wash, each solution included 0.1% igepal. Biotinylated RNAs were eluted twice for 5 minutes each in a 200μL solution of 0.1X MPG containing 5% β-mercaptoethanol. The first elution step was at 25°C and the second was at 65°C. The two eluted fractions were combined and extracted with PCA once and chloroform twice. After extraction, 40μL of 10M NH_4_OAc was added to each tube, and the RNA was precipitated in 70% ethanol.

### Nuclear run-on assay

Nuclear run-ons were performed essentially as previously described [67]. The details are provided in the Expanded View.

### 4SU pulse chase

Following knockdown, cells were treated with 2μM of 4SU for one hour. Afterwards, cells were washed twice with phosphate buffered saline (PBS) containing calcium and magnesium (Sigma-Aldrich), and grown in media lacking 4SU for an additional hour. After the one-hour washout step, we collected 0, 30, 60, and 120 min time points. 40μg of RNA was used as input for a biotinylation and streptavidin selection as described above. Selected RNA was reverse transcribed prior to qRT-PCR analysis. β-actin was used as a loading control for qPCR analysis.

### 4SU nascent RNA capture

The cells were given fresh media 4.5 hours prior to the 4SU treatment, which was necessary for consistent results. Cells were treated with 100 μM of 4SU for five minutes and incorporation was quickly stopped by addition of TriReagent. Sixty micrograms of total RNA was used for biotinylation and streptavidin selection as described above except one additional 1X MPG and one additional no salt wash was performed and both elution steps were done at room temperature.

## Supporting Information

S1 Fig Global analysis if PPD targets. *(A) Left*, Quantitative western blots showing PABPN1 expression levels in the LALA stable cell line as well as knockdowns of PABPN1, UPF1, DIS3 and RRP6. LALA contains a myc-tag. *Right*, RNA-seq traces of PAPα and PAPγ from “total” samples; ACTB is an unaffected transcript shown for reference. As expected PAPα and PAPγ were listed among the most significant downregulated DEGs in the siPAP datasets (S1 Table). Unfortunately, suitable antibodies were not available for PAPα/γ western blot. *(B)* Composite RNA profiles comparing the fold changes from all three datasets of the high-confidence PROMPTs (solid lines) or the entire genome (dotted lines) relative to the TSS. These data are from the second biological replicate whereas those in Fig 1G are from another. *(C)* Scatter plot of DEGs from each of the three datasets using GENCODE lncRNA annotations. Data were analyzed using EdgeR. The log_2_ fold change (FC) is relative to the matched untreated parental cell line using total RNA or nuclear RNA as appropriate. The relative expression in the control lines is plotted on the x-axis as counts per million (CPM). *(D)* Venn diagrams of the up-regulated lncRNA DEGs identified in each of the three samples from (C). *(E)* Venn diagrams of the down-regulated lncRNA DEGs identified in each of the three samples. *(F)* Scatter plot of the high-confidence up-regulated genes in each of the assigned categories. *(G)* Box and whisker plots of the expression levels of the high-confidence PPD target categories. The asterisk and ns are statistically significant differences or not significant, respectively, compared with the entire high-confidence group “DEGs (UP)” (p-value<0.0001, Mann-Whitney test).
(EPS)

S2 Fig Correlation between number of exons and PPD sensitivity. Box and whiskers plot of the fold change in the siPAP total samples compared to the number of exons in the gene. The asterisks denote p-value <0.05 by Mann-Whitney test.
(EPS)

S3 Fig Role of PPD factors in RI-containing transcript decay. *(A)* Cells were fractionated after knockdown with PABPN1 or control siRNAs and RNA was detected by northern blotting (top) and quantitated by Phosphorimager (bottom). The bars are the mean of three biological replicates and the error bars are standard deviation. *(B)* The mRNA isoforms of each of the indicated genes were quantified after detection by northern blot or qRT-PCR as in Fig 4D. The bars are the mean of at least three biological replicates, the error bars are standard deviation, and asterisks denote a P<0.05 using an unpaired Students t-test. *(C)* NRO analysis as in Fig 3E except siRNAs against PAP were tested. No genes showed statistically significant decreases in transcription after PAP knockdown (*n = 4*). *(D)* Northern blot of RNAs from total (T), cytoplasmic (C) or nuclear (N) RNA using an exonic probe that recognizes mRNA and RI-RNA isoforms. Lanes 1–3 are duplicated from Fig 3. Lanes 4–6 are samples that were treated with ActD for 6 hr. The asterisk highlights the fully spliced, but hyperadenylated isoforms. All samples were run on the same gel and are displayed at the same exposure. However, some lanes were cropped for display (vertical dotted line). (E) Live cells were treated for 5 min with 4SU after depletion of PABPN1 (72 hr) or PAP (96 hr) or in a matched control. RNA was harvested, 4SU-containing RNAs were selected and analyzed by qRT-PCR. Because of the brief pulse, most of the selected RNA represents nascent transcripts and therefore reflects transcription rates. Two separate controls (siControl) were performed at 72 and 96 hrs; siPABPN1 and siPAP were normalized to the matched control, but only one is shown for simplicity. Asterisks denote a P<0.05 using an unpaired Students t-test.
(EPS)

S4 Fig Hyperadenylation upon transcription inhibition is a general phenomenon. (A) Total cellular poly(A) tails were examined by northern blot at various time points after flavopiridol, triptolide or DRB treatments for the indicated periods of time. Molecular weight marker (kb) are shown on the left. (B) Hela cells and bone marrow derived primary mouse macrophages were treated with ActD and total cellular RNA was used for bulk poly(A) tail analysis.
(EPS)

S5 Fig PANΔENE-A_35_ is efficiently processed and stabilized by LALA. *(A) Left*, cartoon of the PANΔENE-A_35_ and PANΔENE-AAUAAA RNAs with the positions of the 5´ and 3´ oligonucleotide probes shown (arrows). *Right*, after a 2-hr pulse, RNA was analyzed by hybridizing first to a 3´ probe, which detects the RNAs not cleaved by RNase P and the same blot was subsequently stripped and re-probed with a 5´ probe that detects both cleaved and uncleaved RNAs. The relative amount of RNA not cleaved by RNase P was determined by comparison of the 3´ signal to the 5´ signal. *(B)* Transcription pulse chase of PANΔENE-A_35_ following transfection of pcDNA or LALA, as indicated.
(EPS)

S6 Fig Effects of PPD on PROMPTs and potential premature PAS sites. *(A)* Inspection of four previously published PROMPTs. In each panel the blue is the minus strand and black is the plus strand sequence. ProRBM39 is essentially undetectable in our cells indicating it is either very efficiently degraded in a PPD-independent fashion or it is not transcribed. ProEIF4ENF1 shows modest up-regulation in the LALA samples but little or no effect upon PAP knockdown. ProCCDC93 shows a weak up-regulation in all three samples. ProFOXP4 is up-regulated under all three datasets, but it was not among the high-confidence target list. *(B)* Sequence traces from the five mRNA genes that appear to be prematurely terminated. The controls are gray, while the siPAP total, siPAP nuclear, and LALA samples are in black, orange, and purple, respectively. Note that for APOLD1 and FAM120C, full-length RNAs are generated and these are not affected by PPD inactivation as assessed by the signal on the 3´ UTRs of these RNAs. However, the 5´end signal is enriched in all three treated lanes.
(EPS)

S1 Text Additional materials and methods. Descriptions of plasmid construction and NRO assay.
(DOCX)

S1 Table DEGs identified in this study. This spreadsheet includes all DEGs from all comparisons in this study. See “notes” sheet for a complete description.
(XLSX)

S2 Table High confidence DEG list. These datasets include the upregulated and downregulated genes that were identified in all three datasets.
(XLSX)

S3 Table DEGs identified from GENCODE lncRNAs. This dataset includes all of the DEGs identified using the lncRNA annotations from GENCODE as described in the Materials and Methods.
(XLSX)

S4 Table Estimated size ranges of MAT2A, OGT, and ARGLU1 poly(A) tails +/- 6-hr ActD treatment. Molecular weight markers on northern blots (Fig 4B) were used to estimate the size ranges of poly(A) tails of the specific RNAs. A_0_ refers to the size after RNase H cleavage (Fig 4B), excluding the poly(A) tail.
(DOCX)

S5 Table Primer table. This table lists names and sequences of all primers used in this study.
(XLSX)
