# Supplementary material for: Canonical Poly(A) Polymerase Activity Promotes the Decay of a Wide Variety of Mammalian Nuclear RNAs
Source: PLoS Genet. 2015 Oct 20;11(10):e1005610. doi: 10.1371/journal.pgen.1005610 (PMC4618350; doi:10.1371/journal.pgen.1005610)
Supplement: S1 Text — Descriptions of plasmid construction and NRO assay. (DOCX) [file pgen.1005610.s007.docx]

**SUPPORTING INFORMATION (Bresson et. al, 2015)**

**MATERIALS AND METHODS**

**Plasmid construction**

The TetRP-driven PANΔENE-AAUAAA was generated by digestion of PANΔ115-Bgl (Conrad et al. 2006) with NcoI and EcoRV. The resulting fragment was cloned into TRP-PANΔ79 (Conrad et al. 2007) digested with the same enzymes. TetRP-driven PANΔENE-AAUAAA includes a 115 nt deletion encompassing the 79 nt ENE. In order to generate PANΔENE-A35, we performed PCR on genomic DNA to amplify a stretch of the MALAT1 gene. We cloned the mascRNA element along with 60 nucleotides of downstream sequence in case these were essential for RNase P cleavage. The forward primer included a stretch of 35 adenosine residues in order to generate a short poly(A) tail directly upstream of the predicted cleavage site in the mature RNA. The resulting PCR fragment was cloned into PANΔ115-AAUAAA using the BglII site.

PABPN1-LALA was previously described (Bresson and Conrad 2013). To generate the stable cell line expressing LALA, we used the pcDNA5-FRT/Flp recombinase system (Invitrogen), which allows integration of a given sequence into the genome. We first replaced the CMV promoter of pcDNA5-FRT with TetRP (pcDNA5-FRT-TRP). Next, we digested PABPN1-LALA with BamHI and NotI and cloned the resulting fragment into pcDNA5-FRT-TRP cut with the same restriction enzymes. The resulting plasmid (TRP-LALA) was transfected into 293ATOA cells along with Flp recombinase (pOG44). Stably transfected cells were selected for at least two weeks in 100µg/µL hygromycin and 50ng/mL doxycycline to repress transcription. Thereafter cell stocks were maintained in 50µg/µL hygromycin.

The MS2-only construct (pcNMS2-NLS-Fl) was previously described (Sahin et al., 2010). Using cDNA, we amplified full length PAPOLA (encoding PAPα) with forward and reverse primers containing BamHI and XbaI restriction sites, respectively. Following digestion, the PAPOLA gene was cloned into pNMS2-NLS-Fl cut with the restriction enzymes. To make PAN-6MS2 we first inserted a BglII site 57 nt upstream of the cleavage and polyadenylation site of TRP-PANΔENE (TRP-PANΔ79 BglII-1020). Next, we PCR amplified the six MS2 sites using TetRP-PANΔENE-6MS2 (Stubbs et al. 2012) as a template with primers containing BglII restriction sites. After digestion with BglII, we cloned the resulting fragment into PAN-BglII-1020 cut with the same enzyme.

**Nuclear Run-on assay**

For each knockdown condition, we prepared two fully confluent 10 cm plates of 293ATOA cells (~2x10^7^ cells/knockdown). For the siControl knockdown we prepared two additional 10 cm plates for a no 4SUTP control. The no 4SUTP control was necessary in order to account for nonspecific pulldown. Cells were trypsinized and the cell pellet was lysed in 1mL of hypotonic lysis buffer (HLB) (10mM Tris 7.5, 10mM NaCl, 2.5mM MgCl_2_, 1mM DTT, and 0.5% igepal) for five minutes on ice. The lysate was underlayed with 1mL of HLB supplemented with 25% sucrose, and centrifuged at 600g/5’/4°C in order to isolate nuclei. While on ice, the pelleted nuclei (approximately 60µL in volume) were resuspended in 60µL of 2X transcription buffer (20mM Tris 8.0, 180mM KCl, 10mM MgCl_2_, 50% glycerol, 5mM DTT, 40U RNasIN (Promega), and 250µM each of rATP, rCTP, and rGTP) supplemented with 1µM of either 4SUTP or rUTP (for the no-4SUTP control). 3 µL of a 20% sarkosyl solution was added to each sample with gentle mixing in order to prevent new transcription initiation events. The run-on step was performed for 5 minutes at 30°C before RNA was harvested with TRI reagent. Purified RNA was treated with DNase as described above, with the exception that the reaction was halted with the addition of 25mM EDTA and 15mM EGTA. Following PCA extraction, the RNA was precipitated with ethanol and resuspended to a final concentration of 1mg/mL. 40µL of RNA from each sample was added to 10µL of 1M NaOH, and incubated on ice for precisely 4 minutes in order to hydrolyze the RNA. This step was used in order to better resolve the positions of elongating polymerases. The reaction was stopped with the addition of 61µL of neutralizing solution (833mM Tris 6.8, 500mM NaOAc, and 1µL of glycoblue (Ambion)). RNA was PCA extracted, and biotinylated and selected using magnetic streptavidin beads as described above. Selected RNA was reverse transcribed prior to RT-qPCR analysis.

**REFERENCES**

Bresson SM, Conrad NK. 2013. The Human Nuclear Poly(A)-Binding Protein Promotes RNA Hyperadenylation and Decay ed. G.S. Barsh. *PLoS Genet* **9**: e1003893.

Conrad NK, Mili S, Marshall EL, Shu M-D, Steitz JA. 2006. Identification of a rapid mammalian deadenylation-dependent decay pathway and its inhibition by a viral RNA element. *Mol Cell* **24**: 943–953.

Conrad NK, Shu M-D, Uyhazi KE, Steitz JA. 2007. Mutational analysis of a viral RNA element that counteracts rapid RNA decay by interaction with the polyadenylate tail. *Proc Natl Acad Sci USA* **104**: 10412–10417.

Stubbs SH, Hunter OV, Hoover A, Conrad NK. 2012. Viral factors reveal a role for REF/Aly in nuclear RNA stability. *Mol Cell Biol* **32**: 1260–1270.
