# Supplementary material for: Canonical Poly(A) Polymerase Activity Promotes the Decay of a Wide Variety of Mammalian Nuclear RNAs
Source: PLoS Genet. 2015 Oct 20;11(10):e1005610. doi: 10.1371/journal.pgen.1005610 (PMC4618350; doi:10.1371/journal.pgen.1005610)
Supplement: S4 Table — Molecular weight markers on northern blots (Fig 4B) were used to estimate the size ranges of poly(A) tails of the specific RNAs. A0 refers to the size after RNase H cleavage (Fig 4B), excluding the poly(A) tail. (DOCX) [file pgen.1005610.s011.docx]

| **Transcript** | **-Act D** | **+ Act D (mRNA)** | **+ Act D (RI)** | **A_0_** |
| --- | --- | --- | --- | --- |
| MAT2A | 50-300 | N.D. | 500-700 | 515 |
| OGT | 50-300 | 50-150 | 300-600 | 620 |
| ARGLU1 | 50-600 | 50-200 | 500-800 | 540 |

**Table S4. Approximate size ranges (nt) of MAT2A, OGT, and ARGLU1 poly(A) tails +/- 6-hr ActD treatment.**
